# YTHDF2 exerts tumor-suppressor roles in gastric cancer via up-regulating PPP2CA independently of m^6^A modification

**DOI:** 10.1186/s12575-023-00195-1

**Published:** 2023-03-04

**Authors:** Ying Zhou, Kailing Fan, Ning Dou, Li Li, Jialin Wang, Jingde Chen, Yandong Li, Yong Gao

**Affiliations:** 1 Department of Oncology, Shanghai East Hospital, School of Medicine, Tongji University, 150 Ji-Mo Rd., Shanghai, 200120 China; 2 Department of Oncology, Shanghai East Hospital Ji’an Hospital, Ji’an City, 343000 Jiangxi Province China; 3 School of Medicine, Tongji University, Shanghai, 200120 China

**Keywords:** YTHDF2, Gastric Cancer, Progression, PPP2CA

## Abstract

**Background:**

YTHDF2 is one of important readers of N6-methyladenosine (m^6^A) modification on RNA. Growing evidence implicates that YTHDF2 takes an indispensable part in the regulation of tumorigenesis and metastasis in different cancers, but its biological functions and underlying mechanisms remain elusive in gastric cancer (GC).

**Aim:**

To investigate the clinical relevance and biological function of YTHDF2 in GC.

**Results:**

Compared with matched normal stomach tissues, YTHDF2 expression was markedly decreased in gastric cancer tissues. The expression level of YTHDF2 was inversely associated with gastric cancer patients’ tumor size, AJCC classification and prognosis. Functionally, YTHDF2 reduction facilitated gastric cancer cell growth and migration in vitro and in vivo, whereas YTHDF2 overexpression exhibited opposite phenotypes. Mechanistically, YTHDF2 enhanced expression of PPP2CA, the catalytic subunit of PP2A (Protein phosphatase 2A), in an m^6^A-independent manner, and silencing of PPP2CA antagonized the anti-tumor effects caused by overexpression of YTHDF2 in GC cells.

**Conclusion:**

These findings demonstrate that YTHDF2 is down-regulated in GC and its down-regulation promotes GC progression via a possible mechanism involving PPP2CA expression, suggesting that YTHDF2 may be a hopeful biomarker for diagnosis and an unrevealed treatment target for GC.

**Supplementary Information:**

The online version contains supplementary material available at 10.1186/s12575-023-00195-1.

## Background

In spite of recent developments in treatment modalities such as surgery, chemotherapy, radiation therapy, and targeted drug treatment, gastric cancer (GC) is still the major health burden, with its morbidity ranking fifth and mortality ranking fourth in the world [1]. Meanwhile, GC is also refractory cancer with high incidence and fatality rate in China [2]. Nowadays, progressive genetic and molecular profiling for stomach cancer has uncovered the heterogeneous features at genomic and transcriptional levels, yielding plenty of new information for the potential therapeutic attempt [3, 4]. The information above shows that a greater understanding of stomach cancer could lead to novel treatment strategies, but promising and prognostic biomarkers served as treatment targets remain limited.

RNA N6-methyladenosin (m^6^A), one of the most common modifications of higher eukaryotic messenger RNA (mRNA), has been reported to be reversibly regulated by m^6^A methyltransferase (“writers, such as METTL3 and METTL14), m^6^A demethylases (“erasers”, such as FTO and ALKBH5),as well as m^6^A methylated recognizing proteins (“reader”, such as YTH (YT521-B homology) domain family, composed of YTHDC1-2 and YTHDF1-3). m^6^A modification has been discovered to play crucial roles in many biological functions, like mRNA splicing, localization, translation, output, and stability [5]. Recently, emerging evidence has pointed out that the dysfunction of m^6^A modification and m^6^A-associated proteins may be related to tumor initiation and development [6].

Importantly, the destinies of m^6^A modified mRNAs are dependent on m^6^A methylated binding protein [7]. YTHDF2, a major m^6^A-specific reader, directly binds to the m^6^A-modified motif within the consensus RRACH sequence to promote the degradation of target transcripts [8, 9]. Thus, YTHDF2 is closely associated with many aspects of tumors by influencing the mRNA stability of tumor suppressors or oncoproteins. In lung adenocarcinoma, Li found that YTHDF2 works as an oncogene by promoting AXIN1 mRNA decay and subsequently activating the Wnt/β-catenin pathway [10], nevertheless, Zhao reported that YTHDF2 serves as a tumor suppressor by regulating the TGFβ1/SMAD2/3 signaling pathway mediated by FAM83D (family with sequence similarity 83D) [11]. In addition, in hepatocellular carcinoma, YTHDF2 has been reported to process the decay of m^6^A-methylated mRNAs of EGFR, IL11 and SERPINE2 and inhibit the malignancy and vascular formation [12, 13]. Interestingly, Sheng also demonstrated that YTHDF2 facilitates the 6-phosphogluconate dehydrogenase (6PGD) mRNA translation, but do not affect the degradation of transcripts [14]. In summary, YTHDF2, for the most part, modulates the degradation of mRNAs containing m^6^A modification, but it also exerts m^6^A-independent regulation in cancer development.

YTHDF2 is closely associated with cancer progression, however, little is known about the function of YTHDF2 in GC. In the present work, we demonstrated the abnormal expression of YTHDF2 in this type of cancer and its tumor-suppressor roles. The catalytic subunit of protein phosphatase 2A (PP2A), PPP2CA, might mediate the tumor-suppressor roles of YTHDF2 in an m^6^A-independent manner.

## Results

### YTHDF2 is down-regulated in gastric cancer

**Fig. 1:**
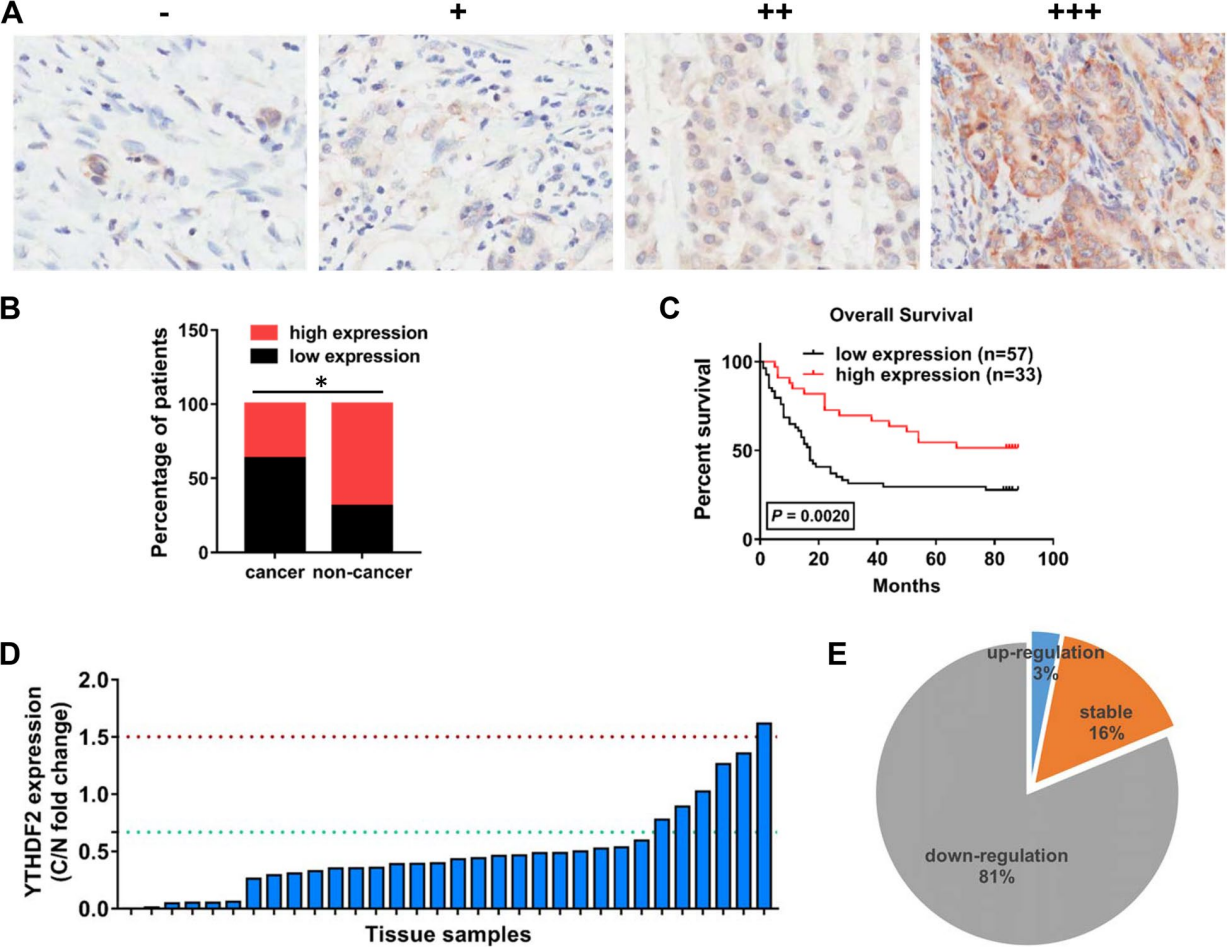
YTHDF2 was aberrantly decreased and negatively related to poor prognosis in GC. **A** Characterization of YTHDF2 protein expression in 90 pairs of gastric tissues with negative (-), weak ( +), moderate (+ +) and strong (+ + +) levels by IHC staining and representative pictures were shown. Magnification: 200 × . **B** The results of IHC staining were shown in the composite column diagram. Low expression: (-) and ( +); high expression (+ +) and (+ + +). **C** Kaplan–Meier plot of different GC patients’ groups divided by YTHDF2 expression level. Survival analysis was evaluated by log-rank test. **D** qRT-PCR results of YTHDF2 mRNA expression in 32 pairs of GC samples. Data are presented as fold changes of YTHDF2 mRNA expression in gastric tissue (C: gastric cancer tissues; N, gastric non-cancer tissues). **E** Percentage of YTHDF2 expression level change in additional 32 pairs of stomach cancer clinical samples

The expression level of YTHDF2 was firstly detected through immunohistochemical staining (IHC) in a GC tissue microarray (TMA), and representative immunohistochemical staining images of YTHDF2 were shown in Fig. 1A, which were ranked from negative (-), weak (+), moderate (+ +) to strong (+ + +). Then, decreased YTHDF2 expression was observed in human GC samples (*n* = 90) compared to paired normal stomach tissues (*n* = 90) (Fig. 1B). Meanwhile, the features shown in Supplementary Table 1 indicated that YTHDF2 expression level was negatively related to AJCC classification (*P* = 0.0005) and tumor size (*P* = 0.0161) of gastric cancer patients. These collective findings highlighted the potential functions of YTHDF2 in gastric cancer development. Moreover, we accessed the relationship between the prognosis and YTHDF2 expression level of 90 GC patients from the above TMA. The results showed that the outcomes of patients with low YTHDF2 expression (*n* = 57) were obviously poorer than the outcomes of patients with high YTHDF2 expression (*n* = 33) as illustrated by the overall survival (OS) analysis (Fig. 1C). Next, to investigate mRNA expression level of YTHDF2 in GC, we collected additional 32 pairs of stomach tissues including GC samples and normal adjacent tissues and detected the mRNA levels of YTHDF2 by qRT-PCR. The GC samples with YTHDF2 increase more than 1.5-fold change were classified into an up-regulated group, and less than 0.667-fold change were categorized as a down-regulated group and (0.667 ~ 1.5)-fold change were divided into a stable group. Compared to normal tissues, the mRNA level of YTHDF2 was much lower in tumor tissues (Fig. 1D), among which 26 cases (26/32, 81.25%) exhibited a decreased expression (less than 0.667-fold change) (Fig. 1E). Taken together, these data demonstrated that YTHDF2 is down-regulated in gastric cancer, and its expression is negatively correlated with the prognosis and progression of GC patients, implicating that YTHDF2 might act as a prospective biomarker in GC.

### YTHDF2 knockdown enhances gastric cancer cell progressionin vitro

**Fig. 2:**
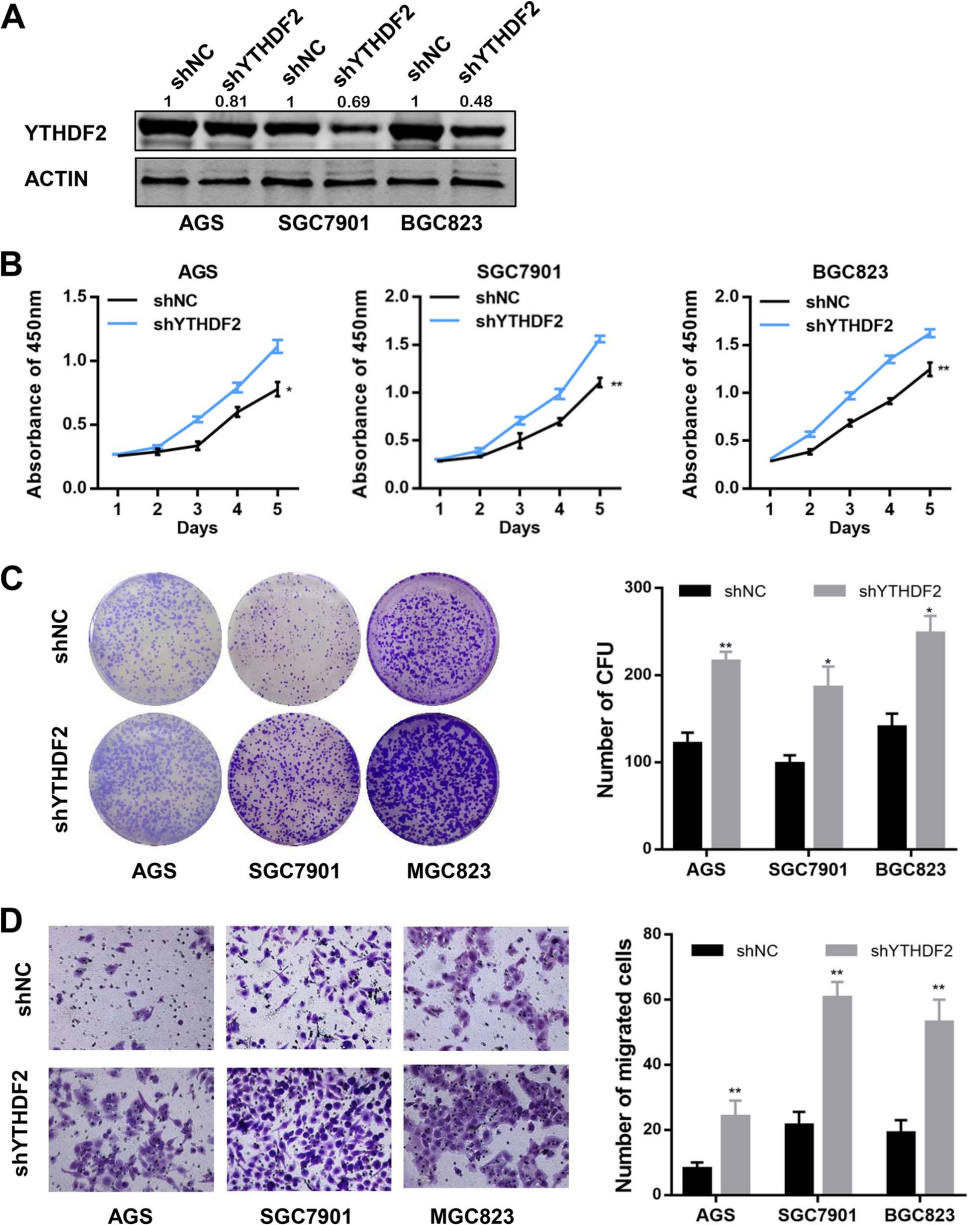
Knockdown of YTHDF2 potentiated gastric cancer cell progression in vitro. **A** Western blot results of YTHDF2 knockdown in GC cells. **B** Cell viabilities were detected using CCK8 assays in stably infected GC cell lines (shYTHDF2 or shNC). **C** Cell growth capacities were determined by colony formation assays in GC cell lines with shYTHDF2 or control shNC. Representative colony-forming pictures (left) and statistical data of the colony-forming number in groups of shYTHDF2 and shNC (right) were shown. **D** Cell migration capacities were conducted by transwell assays in GC cell lines with shYTHDF2 or shNC

To evaluate the biological functions of YTHDF2 in GC cells, we firstly constructed stable cell lines by lentivirus-mediated YTHDF2 knockdown in AGS, SGC7901 and BGC823 cells, and western blot assay was used to confirm the knockdown efficiency (Fig. 2A). The cell proliferation capacity was tested by CCK8 assays and colony-forming assays. The results indicated that YTHDF2 knockdown accelerated the growth rates of AGS, SGC7901, as well as BGC823 cells (Fig. 2B, 2C).​ To further detect the impact of YTHDF2 knockdown on cell migration, transwell chamber assays were carried out in the above stable cell lines, and we found that suppression of YTHDF2 expression augmented the migratory potential of GC cells (Fig. 2D). Additionally, to avoid the off-target effect of RNA interference, we performed CCK8 assay and transwell assay in HGC27 cells transfected with another siRNA against YTHDF2 (siYTHDF2-2) and the results confirmed the function of YTHDF2 knockdown in GC cells (Supplementary Fig. 1). These collective data supported that YTHDF2 knockdown has a crucial effect on GC cell progression in vitro.

### YTHDF2 overexpression significantly hinders GC cell proliferation and migrationin vitro

**Fig. 3:**
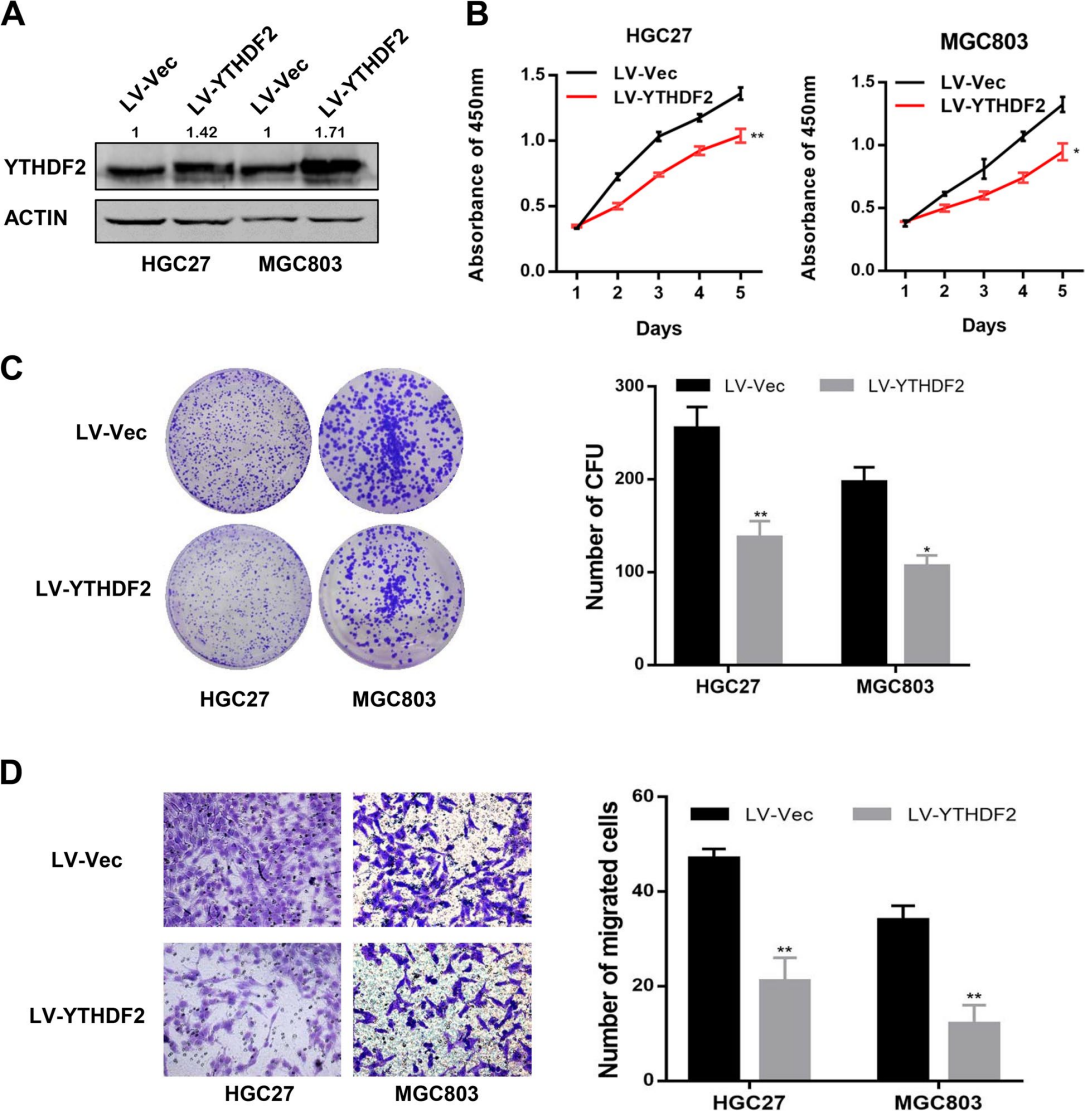
YTHDF2 hindered gastric cancer cell progression in vitro. **A** Western blot results of YTHDF2 overexpression in HGC27, and MGC803 cells. **B** Cell viabilities were detected using CCK8 assays in stably infected GC cell lines (LV-YTHDF2 or control LV-Vec). **C** Cell growth capacities were determined by colony formation assays in GC cell lines with LV-YTHDF2 or control LV-Vec. **D** Cell migration capacities were conducted by transwell assays in GC cell lines with LV-YTHDF2 or LV-Vec

Subsequently, gain-of-function assays were implemented to investigate the effects of YTHDF2 overexpression in gastric cancer cell lines, such as HGC27 and MGC803. YTHDF2 expression was stably overexpressed by infecting these GC cells with lentiviruses (LV-YTHDF2), and overexpression systems for YTHDF2 were successfully established (Fig. 3A). Compared with the control group (LV-vec), YTHDF2 overexpression significantly restrained the proliferation rates of both HGC27 and MGC803 cells via CCK8 assays (Fig. 3B). In accordance with the results of CCK8 assays, the colony formation phenotypes of LV-YTHDF2 groups were markedly smaller and fewer than those of the LV-vec groups (Fig. 3C). Moreover, transwell chamber assays indicated that up-regulation of YTHDF2 strongly attenuated GC cells’ migratory capacities (Fig. 3D). These findings corporately implied that YTHDF2 may play a tumor-suppressive role in GC cells in vitro.

### YTHDF2 has inhibitory effects on neoplasm growth and metastasisin vivo

**Fig. 4:**
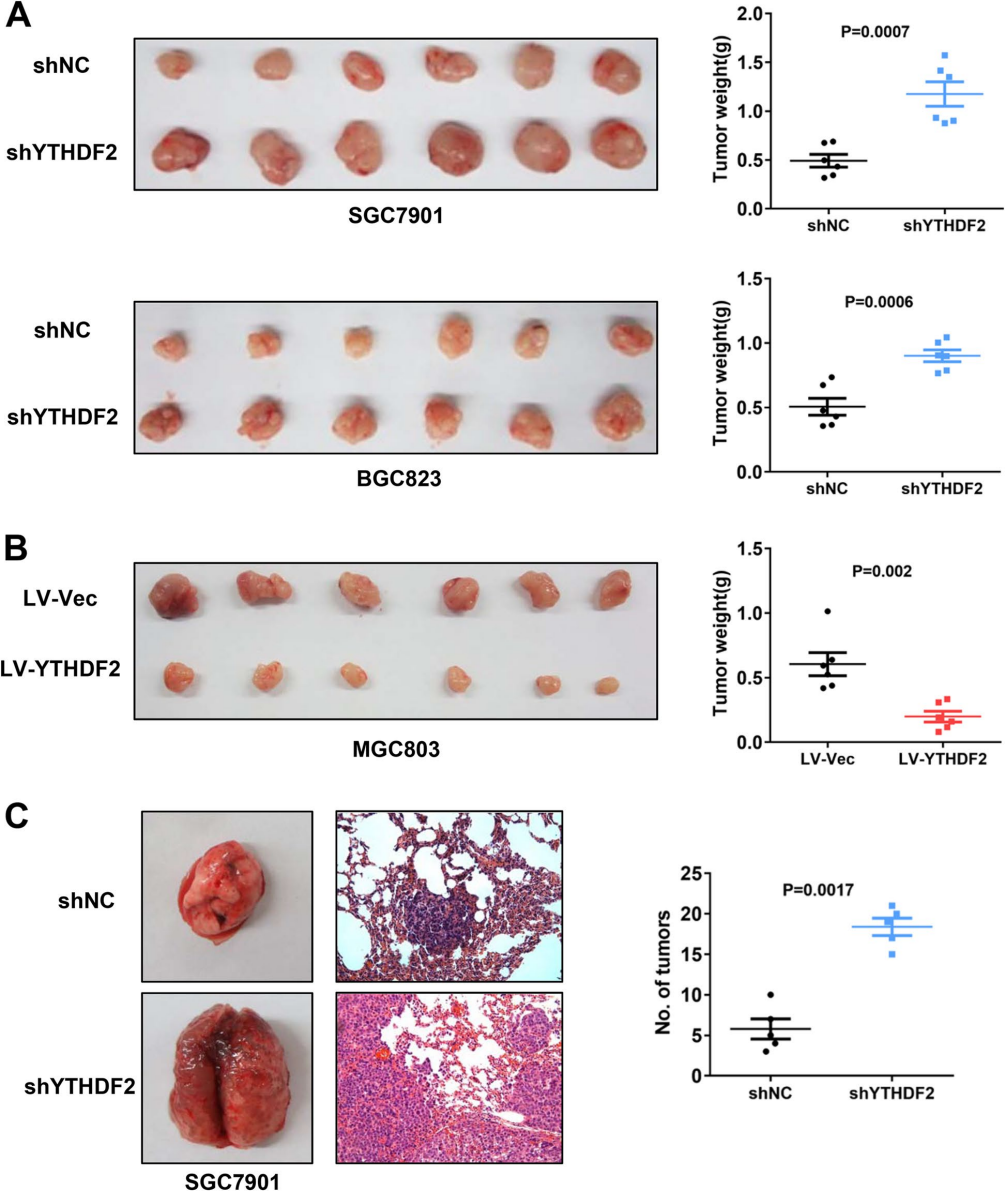
YTHDF2 overexpression attenuated tumorigenicity and metastasis of gastric cancer cell in vivo. **A** YTHDF2 Knockdown increased the carcinogenesis of SGC7901 and BGC823 stable cell lines in nude mice by comparing the sizes and weights of xenografts. **B** Overexpression of YTHDF2 decreased the tumorigenicity of MGC803 stable cell line in nude mice. **C** Knockdown of YTHDF2 promoted pulmonary metastasis of SGC7901 cells. Representative lung tissue images (left), H&E staining pictures of the whole tissues (middle) and statistical data of quantity of metastatic micronodules per lung section(right)

The nude mouse xenograft models were established through subcutaneous injection of gastric cancer cells in which YTHDF2 was stably overexpressed or knocked down, respectively. After four weeks of injection, the mice were sacrificed and the different xenografts were collected and weighed. In line with the in vitro studies, knockdown of YTHDF2 enhanced the volumes and weights of xenografts (Fig. 4A), while overexpression of YTHDF2 led to opposite phenotypes (Fig. 4B). Moreover, the lung metastasis models were generated by tail intravenous injection using GC cells with YTHDF2 stable knockdown. Consistently, the mice of SGC7901/shYTHDF2 group had increased superficial pulmonary metastatic nodules after five weeks of GC cells injection (Fig. 4C). The histological structure of the pulmonary metastatic nodules represented by H&E staining was also performed. These results suggested that YTHDF2 acts as an adverse factor for the gastric cancer progression in vivo.

### YTHDF2 positively regulates PPP2CA expression in an m6A-independent manner

**Fig. 5:**
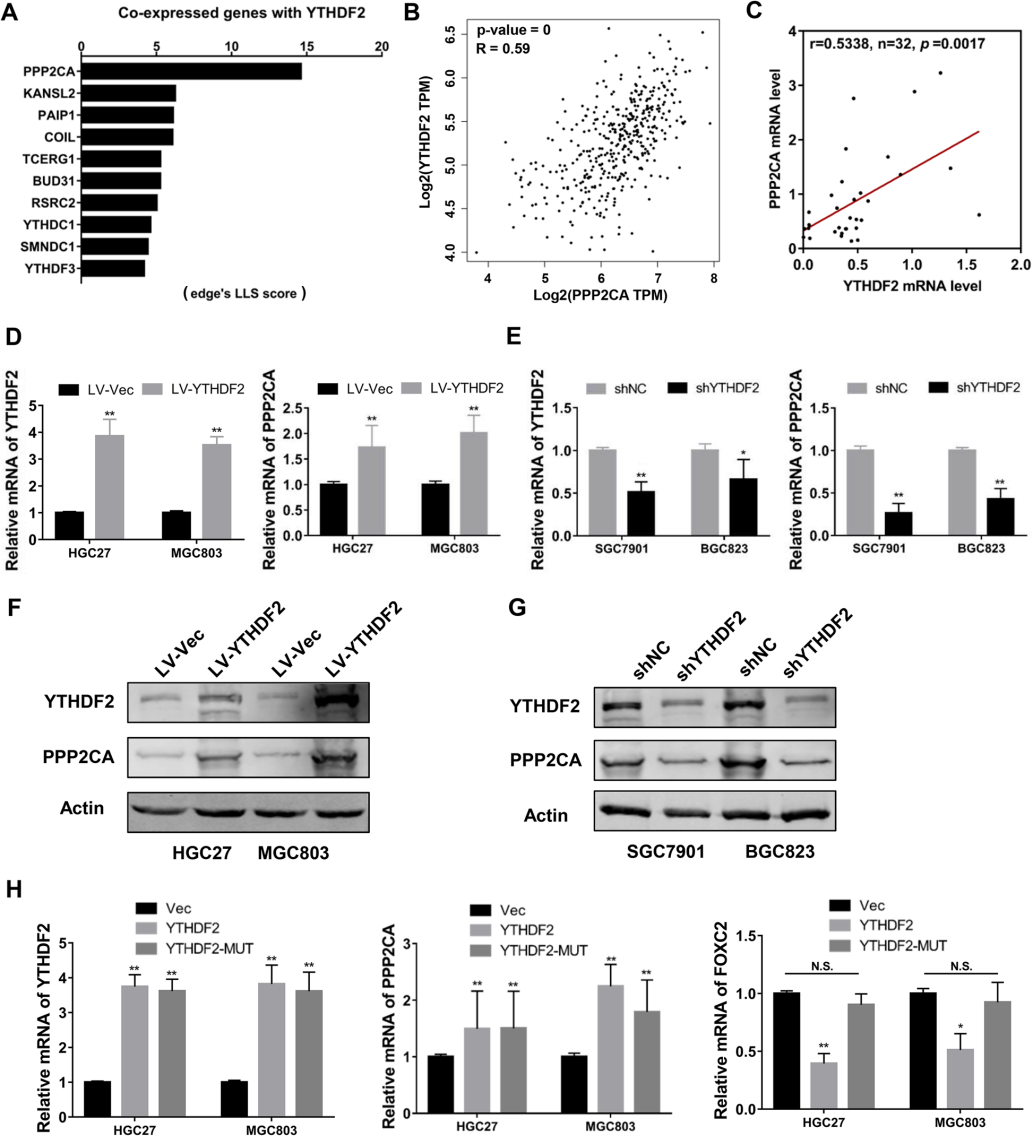
PPP2CA was modulated by YTHDF2 in gastric cancer cells. **A** Data were analyzed by informatics methods from Co-expedia database to investigate co-expression genes of YTHDF2 in stomach cancer. **B** The correlation between YTHDF2 and PPP2CA mRNA expression level was calculated applying GEPIA (*R* = 0.59). C. The expression correlation between YTHDF2 and PPP2CA in 32 pairs of GC samples analyzed from qRT-PCR data. **D**, **E** The mRNA levels of YTHDF2 and PPP2CA in YTHDF2 stably overexpressed or down-regulated GC cells. **F, G** The protein levels of YTHDF2 and PPP2CA in YTHDF2 stably overexpressed or down-regulated GC cells. **H** The mRNA levels of YTHDF2, PPP2CA and FOXC2 in gastric cancer cells where empty vector, YTHDF2-WT and YTHDF2-MUT plasmids were transiently transfected, respectively

To probe into the mechanisms by which YTHDF2 suppresses GC development, a data mining system was used to explore the genes which were positively co-expressed and functionally associated with YTHDF2 in human disease research by the Co-expedia database (https://www.coexpedia.org). Co-expressed genes with YTHDF2 were ranked and shown in Fig. 5A. Simultaneously, the positive correlation between the mRNA levels of YTHDF2 and PPP2CA was represented by GEPIA online visualization tool based on the project of UCSC Xena from TCGA database (Fig. 5B). Similarly, we also observed the positive correlation of YTHF2 and PPP2CA expression in 32 pairs of GC samples according to the qRT-PCR results (Fig. 5C). PP2A (Protein phosphatase 2A) is an indispensable serine and threonine phosphatase that modulates many cellular processes. The PP2Acα (PP2A catalytic subunit Cα, encoded by PPP2CA) is the core enzyme of PP2A, and previous reports revealed that PP2Acα dysfunction plays an important role in the progress of many cancers, such as liver cancer [15] and acute myeloid leukemia [16]. Subsequently, the mRNA expression level of PPP2CA is obviously up-regulated in YTHDF2 overexpression cells (HGC27 and MGC803), whereas down-regulated in YTHDF2 knockdown cells (SGC7901 and BGC823) (Fig. 5D, 5E). Consistently, PPP2CA protein expression was also altered with YTHDF2 protein level (Fig. 5F, 5G), indicating that PPP2CA could be positively regulated by YTHDF2 in GC. On the other hand, we transfected GC cells with a YTHDF2-MUT plasmid, which could not recognize m^6^A modification sites to find out whether the crosstalk between YTHDF2 and PPP2CA relies on m^6^A modification. The result showed that the mutation didn’t affect the regulation of PPP2CA by YTHDF2, but significantly influenced the regulation of FOXC2, a known gene that regulated by YTHDF2 relying on m6A modification (Fig. 5H). These data suggested that YTHDF2-mediated the expression of PPP2CA in an m^6^A methylation-independent manner.

### PPP2CA mediates anti-tumor impacts of YTHDF2 in gastric cancer cells

**Fig. 6:**
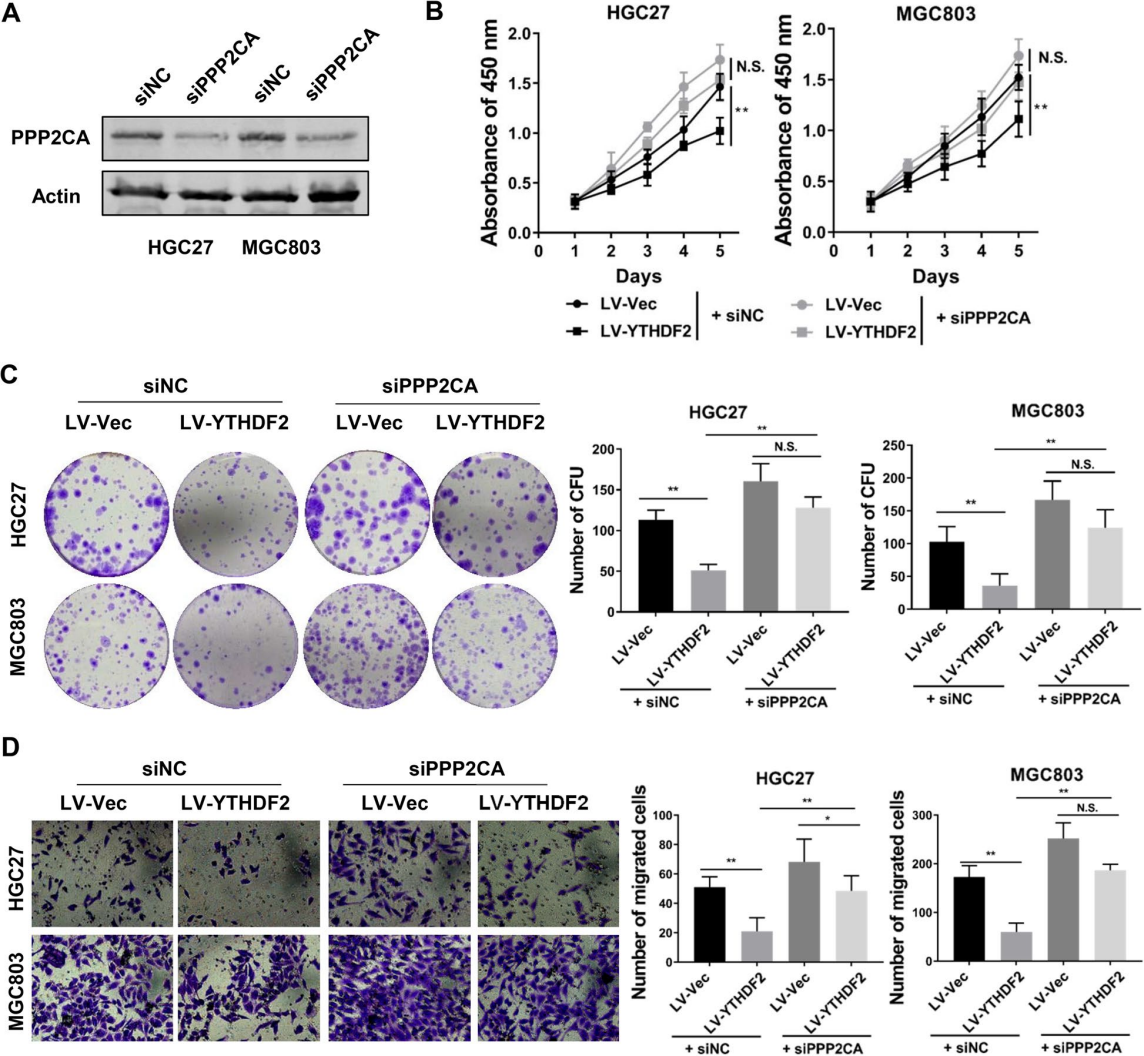
PPP2CA mediated the tumor suppressive functions of YTHDF2 in GC. **A** The knockdown efficiencies of PPP2CA in GC cells transiently transfected with siRNAs against PPP2CA were tested by western blot analysis. **B-D** Cell viabilities and migratory abilities were determined using CCK8 assays (**B**), colony formation assays (**C**) and transwell assays (**D**) in HGC27 and MGC803 cells with stable YTHDF2 overexpression and transfection with PPP2CA siRNA

To further inquire about the relationships between YTHDF2 and PPP2CA, we transiently transfected GC cells with siRNA against PPP2CA, which were authenticated by western blot assays both in HGC27 and MGC-803 cells (Fig. ​6A). As expected, down-regulation of PPP2CA attenuated the proliferation suppression caused by the overexpression of YTHDF2 (Fig. 6B). Similar results were found in colony formation assays for long-term growth and transwell assays for migratory ability of GC cells (Fig. 6C, D, Supplementary Fig. 2A). Furthermore, ectopic expression of PPP2CA could rescue the effect of YTHDF2 knockdown on cell migration (Supplementary Fig. 2B). These results suggested that PPP2CA is a downstream gene of YTHDF2 and a vital contributor to the tumor-suppressive effects of YTHDF2.

### YTHDF2/PPP2CA axis increases cisplatin sensitivity of GC cells

**Fig. 7:**
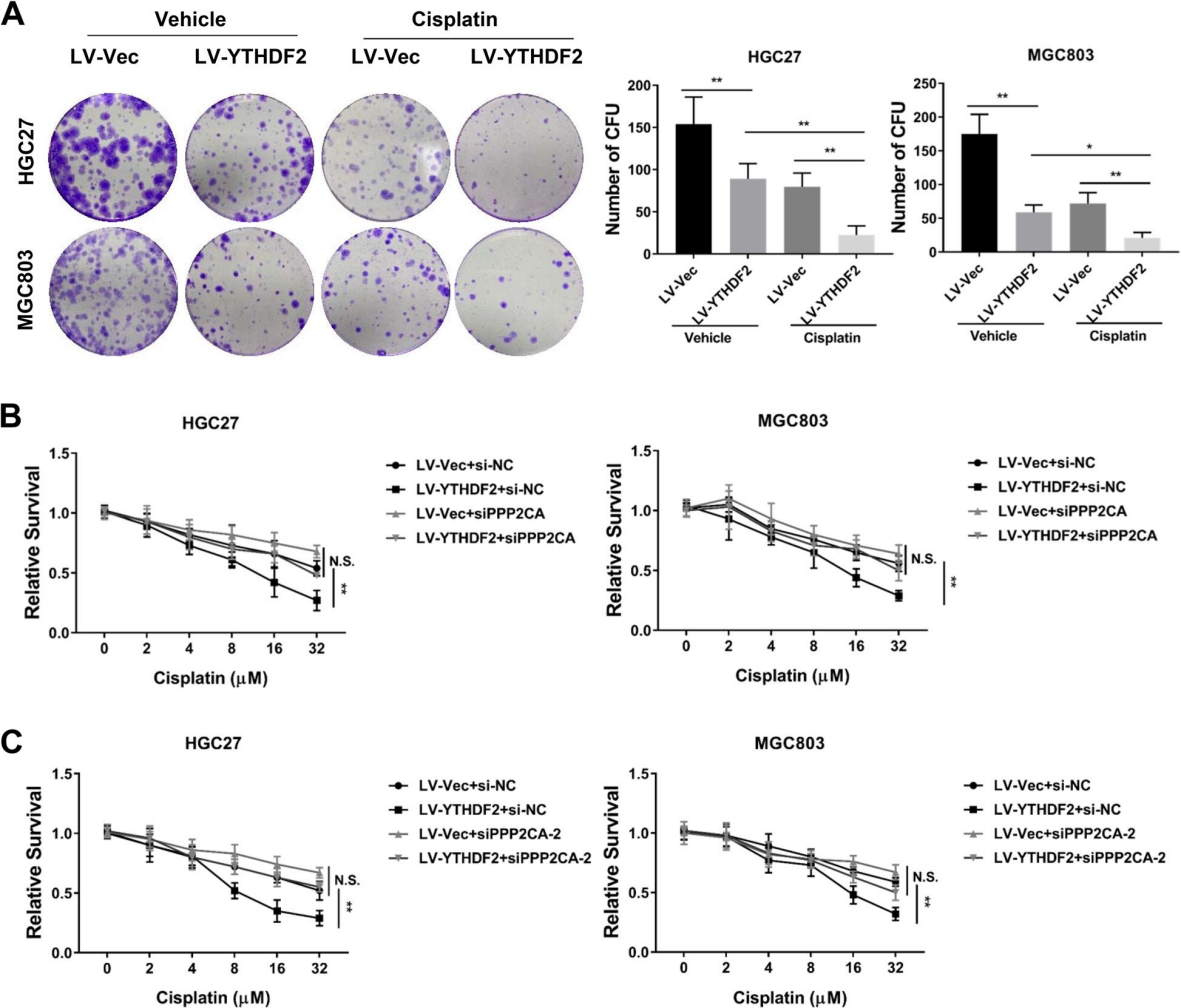
YTHDF2 up-regulation sensitized GC cells to cisplatin via PPP2CA. **A** The capacity of cell growth was detected by colony-forming assays in HGC27 and MGC803 cells with stable YTHDF2 overexpression treated with or without cisplatin (10 μM). **B**, **C** Cell viabilities were detected using CCK8 kit in HGC27 and MGC803 cells with stable YTHDF2 overexpression after being transfected with siPPP2CA (B) or siPPP2CA-2 (C) treated with or without cisplatin as indicated concentrations

Drug tolerance remains a barrier to eligibility for cancer chemotherapy, and resistance to cisplatin is a major handicap to the clinical treatment of late-stage gastric cancer [17]. To test whether YTHDF2 overexpression influenced cisplatin resistance, HGC27 and MGC803 cells with YTHDF2 stable overexpression (LV-YTHDF2) were cultured with cisplatin followed by colony formation assays and we found that YTHDF2 overexpression increased the cisplatin sensitivity of GC cells (Fig. 7A). Considering YTHDF2 positively regulated PPP2CA expression, we tried to elucidate whether the YTHDF2/PPP2CA regulatory axis was involved in the chemotherapy resistance. Knockdown of PPP2CA mitigated GC cells’ cisplatin sensitivity originally caused by YTHDF2 up-regulation as presented by CCK8 analysis (Fig. 7B, C), prompting that YTHDF2/PPP2CA regulatory axis may play a partial role in the drug sensitivity of gastric cancer cells.

## Discussion

Although RNA modifications were uncovered and named in the early 1970s, the significance of eukaryotic RNA modifications has only recently been prioritized by the discoveries of many crucial regulatory proteins and developments in transcriptome-wide m^6^A analysis techniques [18]. As one of the most prevalent and ample eukaryotic RNA modifications, m^6^A modifications occur at different RNA types, including mRNAs, miRNAs and lncRNAs, and play key roles in numerous biological processes, especially in the emergence and development of diverse cancers [19].

Among m^6^A regulators, YTHDF2 was the first explored and the most potent m^6^A “reader” [9]. The expression profiling of YTHDF2 has been found to differ in different types of cancer. Furthermore, YTHDF2 has been reported to exert dual functions in an m^6^A-dependent manner relying on whether the downstream target genes are tumor-promoting or tumor-suppressing. For example, YTHDF2 was increased and functioned as a unique therapeutic target for acute myelocytic leukemia (AML) [20]. In contrast, it was reported that YTHDF2 also had an anti-leukemic activity effect by attenuating the stability of MYC/CEBPA mRNA [21]. Another study revealed that YTHDF2 was down-regulated as a tumor suppressor and inhibited melanoma tumorigenesis [22]. Nevertheless, the study on YTHDF2 in gastric cancer is limited. The current studies suggested that the half-life of PTEN mRNA was shortened by LINC00470-METTL3 axis and relied on the YTHDF2-dependent pathway [23]. Besides, Shen found that YTHDF2 recognized and mediated the m^6^A modification of FOXC2 and restrained gastric cancer cell growth via degrading FOXC2 [24], but which “writer” added the m^6^A modification of FOXC2 mRNA in this study was still unclear. In our study, we disclosed that compared with paired normal adjacent stomach tissues, the expression level of YTHDF2 was frequently down-regulated in gastric cancer tissues, whereas down-regulated YTHDF2 expression was relevant to larger tumor sizes, higher TNM stages and poorer survival status. Using gain-of-function or loss-of-function assays, we identified that YTHDF2 suppressed gastric cancer cell proliferation and migration in vitro and in vivo, supporting a cancer-suppressing function of YTHDF2 in gastric cancer, consistent with previous related studies.

Otherwise, YTHDF2 can directly regulate some mRNAs. As reported by Jin, YTHDF1 and YTHDF2 interacted competitively with YTHDF3 to module YAP expression in lung cancer in an m6A-independent manner [25]. In addition, YTHDF2 could also speed up YAP mRNA degradation through the Argonaute 2 (AGO2) system, inhibiting tumor cell proliferation and metastasis to mitigate disease progression [25]. More importantly, we suggested that YTHDF2 could suppress the progression of gastric cancer via regulating PPP2CA and exert a tumor suppressor effect in an m^6^A-independent manner for the first time.

PP2Ac, the catalytic subunit of PP2A, encoded by PPP2CA, is a ubiquitously expressed protein with a tightly regulatory mode [26]. PP2A has attracted the attention of researchers because of the complexity of its trimeric structure, with its substrate specificity and functional diversity, which enriched the functions of the PP2A holoenzyme [27]. However, PP2A’s core enzyme, which consists of structural subunit A and catalytic subunit C, was indispensable for functional diversity [28]. PP2CA has been reported as a serine/threonine phosphatase involved in multiple cellular processes. It is highly conserved, ubiquitously expressed and tightly controlled. What’s more, PP2Acα dysfunction often caused the loss of PP2A holoenzyme activity, driving deregulation in cellular functions and inducing various diseases [29].

PP2A, as a confirmed tumor suppressor, was verified to be altered or functionally inactivated in many tumors [30, 31]. So far, the basic research about stomach cancer has not involved PP2Acα/PPP2CA except that PP2Acα plays an antineoplastic role through the ATM/METTL3 Axis in Cheng’s research [32]. In our work, we identified PPP2CA as a direct downstream of YTHDF2 by combining a comprehensive analysis of bioinformatics strategies and experimental evidence. PPP2CA reduction greatly increased colony-forming capacity, metastatic properties, and drug resistance of GC cells that were inhibited by YTHDF2 overexpression. Moreover, the up-regulation of mRNA expression level of PPP2CA induced by overexpression of YTHDF2 was also observed in cells transfected YTHDF2-MUT plasmid, which lose the ability to recognition of m^6^A modification sites, thus we verified YTHDF2 positively regulated PPP2CA expression in an m^6^a-independent manner in GC. Interestingly, we found some possible m^6^A modification sites in 3’-UTR of PPP2CA mRNA by SRAMP, a sequence-based m^6^A modification site predictor tool (http://www.cuilab.cn/sramp/). This implies that the regulation of PPP2CA by YTHDF2 is a complex and integrated result and other factors are indispensable for this process. Finally, PPP2CA is a targetable tumor suppressor that can be achieved by PP2A-activating drugs or by antagonists of PP2A inhibitors [33, 34]. In future studies, we will aim to explore whether targeting PP2A or PPP2CA is useful for YTHDF2-mediated anticancer effects.

## Conclusion

Taken together, our research offers novel insights into the novel m^6^A-independent effect of YTHDF2 in GC. We found that PPP2CA may mediate YTHDF2-regulated cell proliferation, migration and chemoresistance. Targeting the YTHDF2/PPP2CA axis may be a potential therapeutic strategy against gastric cancer and YTHDF2 might become a hopeful diagnostic biomarker for this disease in the future.

## Materials and methods

### Human tissue samples and ethics statement

For the immunohistochemistry staining assay, we obtained a gastric cancer tissue microarray (TMA, #HStmA180Su11, Outdo Biotech, Shanghai, China) including 90 paired human GC cancer specimens and matched normal specimens with detailed clinical information. Standard immunohistochemical procedures were carried out by applying specific antibodies against YTHDF2 **(#**24,744–1-AP, Proteintech, Wuhan, China). Two independent pathologists assessed GC patients’ immunohistochemical staining results in a double-blind way [35]. Additional 32 paired GC clinical specimens were obtained at Shanghai East Hospital from patients who signed informed consent before operations and underwent surgical resection. Then the primary GC specimens were immediately snap-frozen at liquid nitrogen and reserved at -80 °C till RNA isolation. This study was approved by the Medical Ethics Committees of Shanghai East Hospital, Tongji University.

### Cell culture

Human GC cell lines, HGC27, MGC803, SGC7901, AGC and BGC823, were obtained from the Shanghai cell bank of the Chinese Academy of Sciences and maintained in Dulbecco’s Modified Eagle’s medium (DMEM, Corning, USA) containing 10% fetal bovine serum (FBS, Corning, USA) and penicillin–streptomycin (100 units/mL, M&C Gene Technology, China) in a humidified incubator at 37℃ with 5% CO_2_.

### Plasmid construction, transfection and siRNA interference

The plasmids of YTHDF2-WT and YTHDF2-MUT were kind gifts from professor Tiebang Kang (Sun Yat-sen University Cancer Center, Guangzhou, China) [12]. To establish stably cell lines with YTHDF2 overexpression, we firstly generated lentiviral particles via co-transfected YTHDF2-WT with pMD2.G and pSPAX2 into 293FT cells as previously described [36]. The shYTHDF2 and shNC lentiviruses were constructed and purchased from GenePharma (China, Shanghai) according to the targeting sequence for YTHDF2: 5-GACCAAGAAUGGCAUUGCA-3 and for Negative control: 5-UUCUCCGAACGUGUCACGU-3. Stable GC cell lines were established by lentivirus infection and puromycin selection (5 μg/mL). The expression of PPP2CA was down-regulated by transiently transfected with small interfering RNA (siRNA) (GenePharma, Shanghai, China): siNC (5’-UUCUCCGAACGUGUCACGUdTdT-3’), siPPP2CA (5’-CAUGGAACUUGACGAUACUdTdT-3’) and siPPP2CA-2 (5’-GGCAGAUCUUCUGUCUACAdTdT-3’). For transient knockdown of YTHDF2 in GC cells, siYTHDF2-2 (5’-GCAGACTTGCAGTTTAAGTAT-3’) was also synthesized by GenePharma, Shanghai, China. A FLAG-tagged PPP2CA overexpression plasmid, pENTER-PPP2CA (NM_002715), was obtained from Vigene Biosciences, Shandong, China. Cell transfection with siRNAs or plasmids was performed with Lipofectamine 3000 (Invitrogen, USA) according to the manufacturer’s instructions.

### RNA extraction and quantitative real-time polymerase chain reaction (qRT-PCR)

TRIzol reagents (Sigma-Aldrich, Merck KGaA, Germany) were used to isolate total RNAs from gastric cancer cells. The cDNA was reverse transcribed by applying the Primescript™ RT Reagent kit (Takara Shuzo, Kyoto, Japan). SYBRGreen reagents (Takara Shuzo, Kyoto, Japan) were applied to perform the qPCR analysis on an ABI QuantStudio™ 6 Flex system. The primer sequences were used: YTHDF2-F: 5′-TAGCCAGCTACAAGCACACCAC-3′, YTHDF2-R:5′-CAACCGTTGCTGCAGTCTGTGT-3′; PPP2CA-F: 5′-GTTCGTTACCGTGAACGCATC-3′, PPP2CA-R: 5′-TGGCGAGAGACCACCATGT-3′; FOXC2-F: 5′-CGCCTAAGGACCTGGTGAAG-3′; FOXC2-R: 5′-GGAAGCGGTCCATGATGA -3′; β-actin (endogenous control)-F: 5′-CCTGGCACCCAGCACAATG-3′, β-actin (endogenous control)-R: 5′-GGGCCGGACTCGTCATACT-3′. The relative expression was normalized to *β*-actin by 2^−ΔΔCt^ method.

### Cell proliferation assay

For cell growth and viability assay, 3,000 to 4,000 GC cells were planted in a fresh 96-well plate and maintained in culture medium for five days. Cell Counting kit-8 (10 µl CCK8, Dojindo, Kumamoto, Japan) was dropped to 96-well plate and incubated at 37 °C incubator for 75 min every 24 h. Subsequently, absorbance at 450 nm wavelength was measured using SpectraMax M5 (Molecular Devices, USA).

### Colony formation assay

Two thousand GC cells were planted in 6-well plates and maintained for about 14–21 days. After being washed twice with PBS, cells were fixed and stained using 4% paraformaldehyde and 0.5% crystal violet for 15 min.

### Transwell assay

Thirty-thousand GC cells were plated in the top chamber (Corning, US) in culture medium without serum in 24-well plates. The lower chamber was filled with 10% FBS culture medium. After 24-36 h incubation, cells were fixed and stained in 4% paraformaldehyde and 1% crystal violet for 15 min.

### Western blot assay

Whole-cell extracts were collected and lysed on ice in lysis buffer with protease and phosphatase inhibitors (Roche, Indianapolis, IN), and the concentration of protein was measured by BCA Assay Kit (Thermo Fisher Scientific, US). 20 μg target proteins were separated by SDS-PAGE (polyacrylamide gel) and then transferred onto NC (nitrocellulose) membranes (Millipore, US). The NC membrane was incubated for 1.5 h overnight at 4 °C or at room temperature with the primary antibodies at recommended concentration incubated. Then the NC membrane was incubated for 1 h at room temperature with the fluorescent-tagged secondary antibody. Primary antibodies for immunoblotting were as follows: YTHDF2 (1:500, **#**24744–1-AP, Proteintech, Wuhan, China), FLAG (1:1000, **#**66008–4-Ig, Proteintech, Wuhan, China), β-actin (1:500, #81178, Santa Cruz Biotechnology), PPP2CA (1:1000, #2038, Cell Signaling Technology, MA, USA).

### Animal experiments

To detect the ability of tumorigenicity, we established the xenograft model of gastric cancer in BALB/c nude mice (18-20 g, 4 ~ 6-week-old), which was obtained from SLAC Laboratory Animal Company. We injected 2 × 10^6^ stably infected GC cells subcutaneously into each dorsal flank of mice (*n* = 6). After 4 weeks, each histologically intact tumor was removed, weighed and photographed. To construct the model of mice pulmonary metastasis, we injected 2 × 10^6^ stably infected GC cells into the tail vein of the mice (*n* = 5). After 5 weeks, the nude mice of pulmonary metastasis model were sacrificed, we collected the whole pulmonary tissues and calculated and averaged the number of metastatic nodules per lung section. The existence of pulmonary metastases was assessed by microscopy with hematoxylin/eosin(H&E) staining.

### Statistical analysis

Data acquired from each experiment were represented as mean ± standard deviation (SD) from at least 3 independent experiments and analyzed using GraphPad Prism software 8.0. Relationships between YTHDF2 expression and clinicopathological characteristics were calculated by χ2 test. Student’s t-test and one-way ANOVA were carried out to calculate the difference comparison. *P* < 0.05 was served to show statistical significance. *P < 0.05, **P < 0.01, N.S. not statistically significant.

## Supplementary Information

**Additional file 1: Supplementary Fig. 1.** YTHDF2 knockdown by siYTHDF2-2 promoted GC cell proliferation and migration. A Western blot result showing the knockdown efficiency of YTHDF2 in HGC27 cells. B YTHDF2 knockdown promoted cell viability of HGC27 cells via CCK8 assay. C YTHDF2 knockdown promoted HGC27 cell migration via transwell assay.

**Additional file 2: Supplementary Fig. 2.** The functional link between YTHDF2 and PPP2CA in GC cells. A Cell migration capacity was determined by transwell assay in YTHDF2 overexpression cells transfected with siPPP2CA-2 and siNC. Western blot confirmed the knockdown efficiency of PPP2CA. B PPP2CA overexpression suppressed the phenotype induced by YTHDF2 knockdown in AGS cells via transwell assay. FLAG-tagged PPP2CA was detected by western blotting.

**Additional file 3: Supplementary Table 1.** The association of YTHDF2 expression with clinicopathological features of GC patients.

## Data Availability

All data analyzed and generated in this study are included in this published article.

## Abbreviations

GC: Gastric cancer
TCGA: The cancer genome atlas
TMA: Tissue microarray construction
IHC staining: Immunohistochemical staining
m^6^A: N6-methyladenosine modification
