# Supplementary figures and images for: YTHDF2 exerts tumor-suppressor roles in gastric cancer via up-regulating PPP2CA independently of m6A modification
Source: Biol Proced Online. 2023 Mar 4;25:6. doi: 10.1186/s12575-023-00195-1 (PMC9985201; doi:10.1186/s12575-023-00195-1)

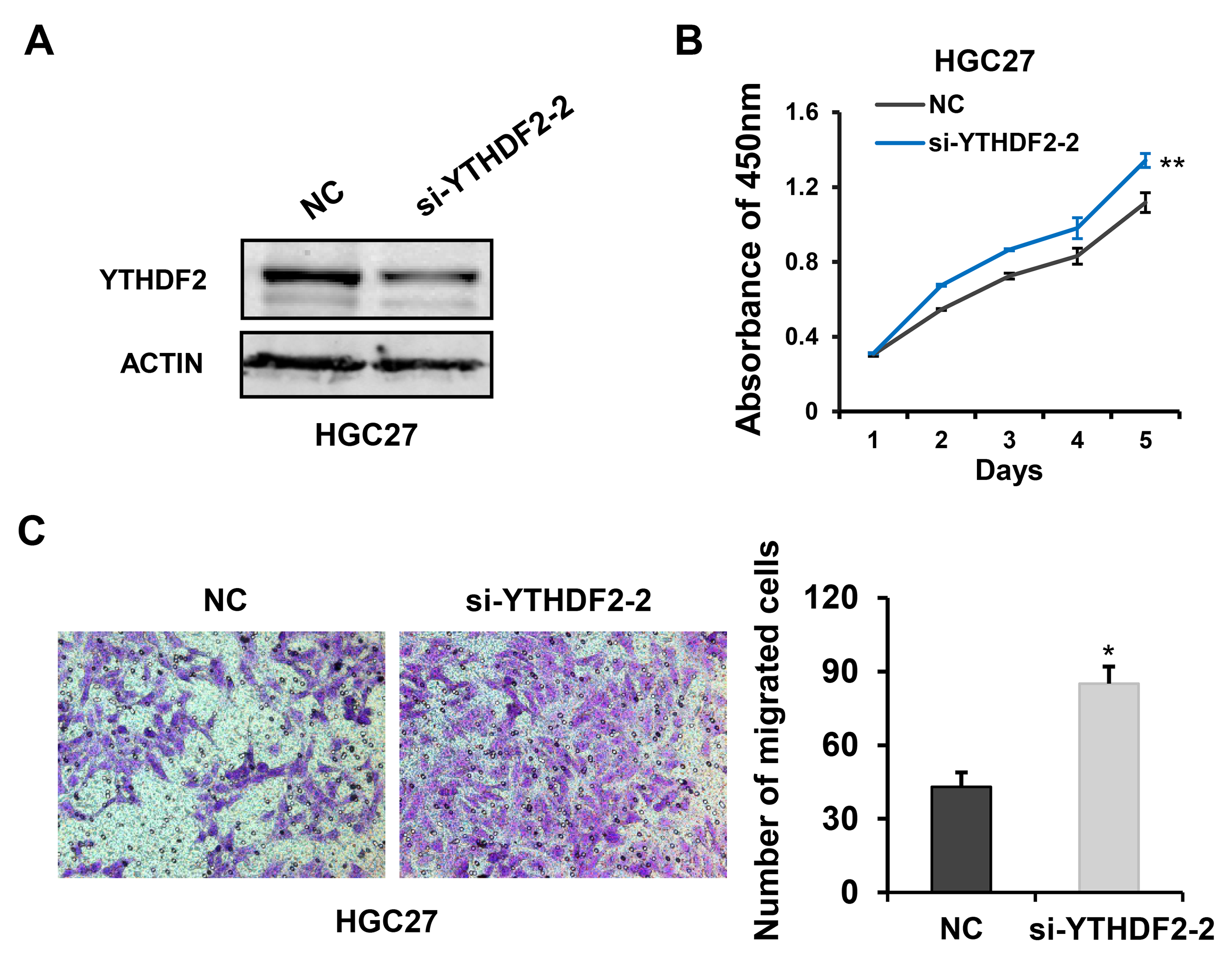

Supplement: Supplementary file 1 — Additional file 1: Supplementary Fig. 1. YTHDF2 knockdown by siYTHDF2-2 promoted GC cell proliferation and migration. A Western blot result showing the knockdown efficiency of YTHDF2 in HGC27 cells. B YTHDF2 knockdown promoted cell viability of HGC27 cells via CCK8 assay. C YTHDF2 knockdown promoted HGC27 cell migration via transwell assay. [file 12575_2023_195_MOESM1_ESM.tif]

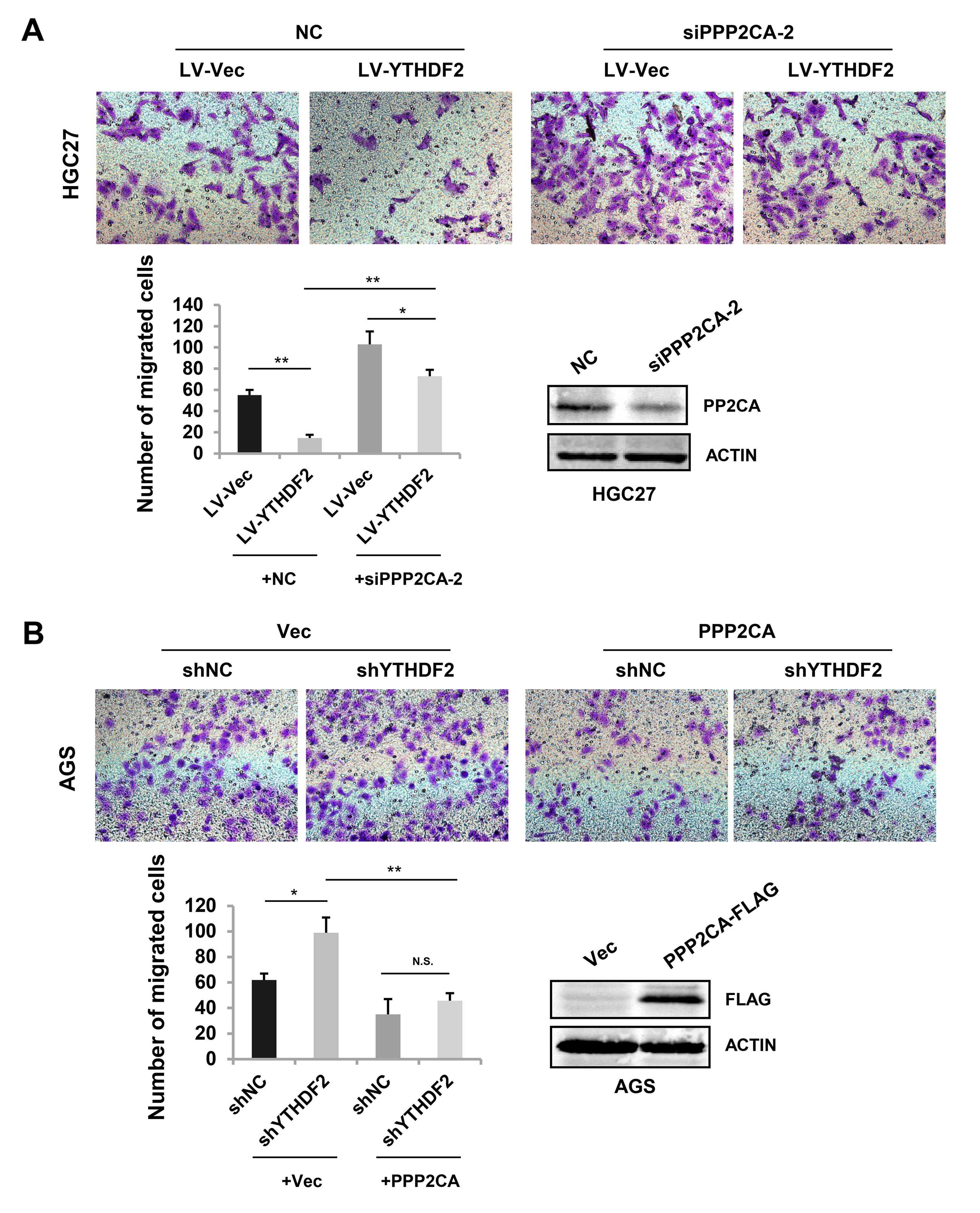

Supplement: Supplementary file 2 — Additional file 2: Supplementary Fig. 2. The functional link between YTHDF2 and PPP2CA in GC cells. A Cell migration capacity was determined by transwell assay in YTHDF2 overexpression cells transfected with siPPP2CA-2 and siNC. Western blot confirmed the knockdown efficiency of PPP2CA. B PPP2CA overexpression suppressed the phenotype induced by YTHDF2 knockdown in AGS cells via transwell assay. FLAG-tagged PPP2CA was detected by western blotting. [file 12575_2023_195_MOESM2_ESM.tif]
