# Supplementary material for: YTHDF2 exerts tumor-suppressor roles in gastric cancer via up-regulating PPP2CA independently of m6A modification
Source: Biol Proced Online. 2023 Mar 4;25:6. doi: 10.1186/s12575-023-00195-1 (PMC9985201; doi:10.1186/s12575-023-00195-1)
Supplement: Supplementary file 3 — Additional file 3: Supplementary Table 1. The association of YTHDF2 expression with clinicopathological features of GC patients. [file 12575_2023_195_MOESM3_ESM.docx]

| Supplementary Table 1:The association of YTHDF2 expression with clinicopathological features of GC patients | | | | |
| --- | --- | --- | --- | --- |
|  | No. of cases (n = 90) | Negative and weak staining (n = 57) | Moderate and strong staining (n = 33) | P value |
| Age (years) |  |  |  |  |
| >60 | 57 | 35 | 22 | 0.6176 |
| **≤**60 | 33 | 22 | 11 |  |
| Gender |  |  |  |  |
| Male | 61 | 38 | 23 | 0.7669 |
| Female | 29 | 19 | 10 |  |
| Tumor size |  |  |  |  |
| >5cm | 58 | 42 | 16 | 0.0161 |
| **≤**5cm | 32 | 15 | 17 |  |
| Differentiation |  |  |  |  |
| Well&moderate-differentiated | 34 | 19 | 15 | 0.2531 |
| Poorly differentiated | 56 | 38 | 18 |  |
| AJCC classification |  |  |  |  |
| I ,II | 36 | 15 | 21 | 0.0005 |
| III, IV | 54 | 42 | 12 |  |
